# Absence of P2Y_2_ Receptor Does Not Prevent Bone Destruction in a Murine Model of Muscle Paralysis-Induced Bone Loss

**DOI:** 10.3389/fendo.2022.850525

**Published:** 2022-05-26

**Authors:** Ankita Agrawal, Maria Ellegaard, Kristian Agmund Haanes, Ning Wang, Alison Gartland, Ming Ding, Helle Praetorius, Niklas Rye Jørgensen

**Affiliations:** ^1^ Department of Clinical Biochemistry, Copenhagen University Hospital Rigshospitalet, Glostrup, Denmark; ^2^ Department of Clinical Experimental Research, Copenhagen University Hospital Rigshospitalet, Glostrup, Denmark; ^3^ The Mellanby Centre for Musculoskeletal Research and The Department of Oncology and Metabolism, The University of Sheffield, Sheffield, United Kingdom; ^4^ Department of Orthopedic Surgery and Traumatology, Odense University Hospital, & Department of Clinical Research, University of Southern Denmark, Odense, Denmark; ^5^ Department of Biomedicine, Aarhus University, Aarhus, Denmark; ^6^ Institute of Clinical Medicine, Faculty of Health and Medical Sciences, University of Copenhagen, Copenhagen, Denmark

**Keywords:** P2Y_2_ receptor, bone, skeletal unloading, botulinum toxin, skeletal reloading

## Abstract

Increased incidence of bone fractures in the elderly is associated with gradual sarcopenia. Similar deterioration of bone quality is seen with prolonged bed rest, spinal cord injuries or in astronauts exposed to microgravity and, preceded by loss of muscle mass. Signaling mechanisms involving uridine-5′-triphosphate (UTP) regulate bone homeostasis *via* P2Y_2_ receptors on osteoblasts and osteoclasts, whilst dictating the bone cells’ response to mechanical loading. We hypothesized that muscle paralysis-induced loss of bone quality would be prevented in P2Y_2_ receptor knockout (KO) mice. Female mice injected with botulinum toxin (BTX) in the hind limb developed muscle paralysis and femoral DXA analysis showed reduction in bone mineral density (<10%), bone mineral content (<16%) and bone area (<6%) in wildtype (WT) compared to KO littermates (with <13%, <21%, <9% respectively). The femoral metaphyseal strength was reduced equally in both WT and KO (<37%) and <11% in diaphysis region of KO, compared to the saline injected controls. Tibial micro-CT showed reduced cortical thickness (12% in WT vs. 9% in KO), trabecular bone volume (38% in both WT and KO), trabecular thickness (22% in WT vs. 27% in KO) and increased SMI (26% in WT vs. 19% in KO) after BTX. Tibial histomorphometry showed reduced formation in KO (16%) but unchanged resorption in both WT and KO. Furthermore, analyses of DXA and bone strength after regaining the muscle function showed partial bone recovery in the KO but no difference in the bone recovery in WT mice. Primary osteoblasts from KO mice displayed increased viability and alkaline phosphatase activity but, impaired bone nodule formation. Significantly more TRAP-positive osteoclasts were generated from KO mice but displayed reduced resorptive function. Our data showed that hind limb paralysis with a single dose of BTX caused profound bone loss after 3 weeks, and an incomplete reversal of bone loss by week 19. Our findings indicate no role of the P2Y_2_ receptor in the bone loss after a period of skeletal unloading in mice or, in the bone recovery after restoration of muscle function.

## Introduction

Limiting bone resorption and regenerating bone tissue are treatment goals in catabolic diseases affecting the skeleton. Applying mechanical forces to the bone, such as during physical activity, serve as cues to activate bone formation pathways and increase bone strength through bone remodeling. Thus, exercise is recommended to prevent osteoporosis (1, 2). Opposingly, the absence of physical stimuli negatively affects the bone health by impeding the achievement of peak bone mass and maximal structural strength. Bone deterioration, such as that seen with advanced age, is characterized by low bone mass and micro-architectural alteration of the bone structure; leading to an increased incidence of low- trauma fractures, chronic pain, extensive disability and morbidity in the elderly population (3). Similar bone fragility is also seen after prolonged bed rest, in sedentary individuals such as in patients with spinal cord injuries, paralysis and epilepsy (4, 5) and in astronauts after spaceflight (6). The reduced muscle contractions with ageing (weakening of internal loading) or the lack of ground reaction forces (reduced gravitational loading) reduces the critical mechanical stimulus to the bone, a process known as unloading.

Unloading leads to decreased bone formation and compromises the communication between the bone-cells (7). Unloading uncouples the tightly coordinated processes of bone formation and bone resorption resulting in reduced osteoblast activity and an increased osteoclast activity. This uncoupling results in a net loss of bone mass and reduced bone strength. The adaptive responses within the bones are highly dependent on the strain from the muscles on the bone, as evidenced during sarcopenia characterized by a progressive and general loss of muscle mass (8). Skeletal muscle contraction releases classical myokines such as irisin, myostatin, fibroblast growth factor- 21 and leptin; with direct effects on osteoblast and osteoclast activity [for review see (9)]. Therefore, therapeutic approaches targeting paracrine signaling from muscle have a potential to enhance bone quality during unloading. The metabolome involved in the muscle- bone crosstalk is populated not only by the many cytokines and growth factors but also extracellular nucleotides with crucial roles in muscle and bone crosstalk.

Mechanical loading of the bone increases local adenosine triphosphate (ATP) concentrations which acts as a signaling molecule [for review see (10)]. Mechanical loading also stimulates uridine triphosphate (UTP) release from non-secretory cells such as osteoblasts and osteocytes (11–16). Both ATP and UTP regulate bone turnover by activating P2Y purinergic receptor 2 (P2Y_2_ receptor) (17), but UTP is slightly more potent than ATP at the human P2Y_2_ receptor with pEC50 of 8.1 and 7.1, respectively (18). The concentration of UTP varies during the physiological and pathological strain on the tissue (19) and activation of the P2Y_2_ receptor regulates a spectrum of functions in the bone microenvironment, making purinergic agents a subject of exciting translational investigations (20).

Activation of P2Y_2_ receptor and coupling to Gq leads to production of phospholipase Cβ (PLCβ), and subsequent IP_3_- dependent Ca^2+^ release from intracellular Ca^2+^ stores and activation of key transcription factors for bone cell differentiation (13, 16, 21). *In vitro* studies from Arnett and colleagues indicate that low concentrations of ATP and UTP strongly inhibit osteoblastic mineralization and alkaline phosphatase activity (22). *In vivo*, 8- week-old male P2Y_2_ receptor knock out (KO) mice show a 9% increase in femoral bone mineral content (BMC) and 17% increase in tibial bone mineral content (BMC) compared to the wild type (WT) mice (C57Bl/6) (23) confirming the anti-osteogenic effect of P2Y_2_ receptor. In a fracture cohort characterized by a prevalence of osteoporosis, a presumed loss of function single nucleotide polymorphism in the gene for the human P2Y_2_ receptor (*P2RY2*, Leu46Pro) is associated with an increase in total hip, lumbar spine and femoral neck bone mineral density (BMD) and a reduced risk of osteoporosis by approximately 30- 40% (24). In contrast, Xing et al. find an osteopenic phenotype in 17-week-old male P2Y_2_ receptor KO mice (SV129), with significantly decreased femoral bone volume and thickness in the trabecular and cortical compartments (25). So far, the evidence indicates the P2Y_2_ receptor as both, anti-osteogenic and anti-catabolic and the discrepancies in the observed bone phenotype can be attributed to the different genetic backgrounds or potential passenger mutations between these P2Y_2_ receptor KO models. To eliminate the influence of the genetic background, we generated BALB/cJ P2Y_2_^-/-^ (KO) and compared with littermate BALB/cJ P2Y_2_^+/+^ (WT) to clarify the role of the P2Y_2_ receptor on bone homeostasis.

In the present study, we additionally examine if P2Y_2_ receptor deletion prevents bone loss after a period of skeletal unloading in mice. Female mice were selected due to their higher rate of bone loss compared to the males. Hindlimb muscle paralysis, using botulinum toxin (BTX), was assessed by digit abduction score (DAS); while bone alterations were determined by DXA, bone strength measurements and microarchitectural CT analyses after 3 weeks. Control mice receiving saline were used to compare BTX induced bone alterations in both WT and KO mice. Any recovery of bone degradation was also determined in both WT and KO mice after 19 weeks of BTX injection. Primary osteoblasts and osteoclasts were differentiated from WT and KO mice to determine the cellular dynamics of P2Y_2_ receptor - mediated bone homeostasis. P2Y_2_ receptor mRNA expression and agonist-evoked intracellular calcium responses were also measured in the cells from WT and KO mice.

## Materials And Methods

### Mice Colony Breeding and Welfare

The P2Y_2_ receptor KO mouse was originally generated by Dr. B Koller, Denmark on a B6D2/SV129 background and brought in house as KO breeder pairs. The P2Y_2_ receptor KO mice were crossed with BALB/cJ strain for >10 generations, generating the BALB/cJ P2Y_2_^+/+^ (WT) and BALB/cJ P2Y_2_^-/-^ (KO) mice for the study. Pups (< 21 days of age) from heterozygous breeding pairs were ear marked and DNA was extracted from tail biopsies for genotyping using the previous primers and method (26). All mice were housed in the same environmentally controlled conditions with a 12- hour light-dark cycle at 23°C. 55 ± 10% humidity with free access to altromin 1314, cereal-based rodent diet (Lage, Germany) and water ad libitum in makrolon type 3 cages (up to 8 mice/cage). Litter mate homozygote WT and KO were selected for all experiments and mice were euthanized at time points as indicated. All procedures complied with Danish animal welfare regulations (Dyreforsoegstilsynet, Copenhagen, Denmark) and were approved under License number: BTX 2012-15-2934-00148. Results from the study are reported according to the ARRIVE guidelines.

### Experimental Design

**Figure 1:**
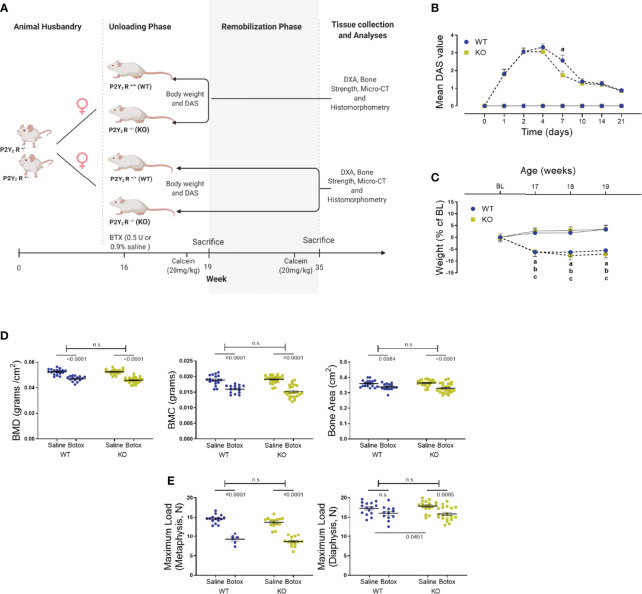
**(A)** The study design to show the unloading (weeks 17 – 19) and remobilization (weeks 20 – 35) phases in BTX injected 16-week old female BALB/cJ mice. Image created by Biorender.com. **(B)** Digit abduction score (DAS) assay to confirm BTX- induced muscle paralysis in both WT and KO mice from day 1 of BTX injection (dotted line) compared to the saline injected mice (solid line). Disability was monitored for 21 days to show the mean peak DAS values at day 4 in both WT and KO. BTX KO, a lower DAS value at day 7 in BTX KO compared to BTX WT (a, P < 0.05) and recovery of muscle function in both WT and KO at the end of 21 days. Statistical significance at day 7 was tested using Student’s unpaired t- test, data shows mean ± SEM of n = 15- 16 mice (WT) and 18- 19 mice (KO) in each treatment condition. **(C)** Weight changes during the unloading phase, expressed as a percentage change from baseline body weight (BL, 16-week old) in WT and KO mice injected with either saline (solid line) or BTX (dotted line). Significant body weight reduction after BTX (a, P < 0.05 in WT and b, P < 0.05 in KO) compared to the saline controls and, in BTX KO compared to the BL (c, P < 0.05). Statistical significance was tested using 2- way ANOVA with Tukey’s test for multiple comparisons. Data shows mean ± SEM of n = 16 mice (WT) and 19 mice (KO) in each treatment group. **(D)** DXA assessments of femoral bone mineral density (BMD, grams/cm^2^), bone mineral content (BMC, grams) and bone area (cm^2^) and, **(E)** femoral maximum load (N), at metaphysis and diaphysis in both WT and KO after BTX- induced muscle paralysis. Statistical significance was tested with either Student’s unpaired t-test/Mann-Whitney test (effect of KO) or, 2-way ANOVA (effect of BTX and KO) with Tukey’s test for multiple comparisons; n.s., no significance. Data shows mean ± SEM of n = 15-18 mice (WT) and 23-24 mice (KO) for DXA and n= 6-15 mice (WT) and 15-19 mice (KO) for bone strength.

Female 16-week-old WT and KO littermates were weighed and randomized into treatment groups. Anesthesia was 1 mL/100 g s.c. mixture of Hypnorm (fentanyl 0.315 mg/ml; fluanisone 10 mg/ml) and midazolam (5 mg/ml) (1:1, v/v) diluted with equal part water. BTX (Allergan Inc.) was reconstituted in 0.9% saline and 0.5 U/mouse solution (20 μl) was injected into the quadriceps and calf muscle of the right leg using an insulin syringe and a 30G needle (BD Medical, France). The contralateral leg remained untreated but was not used as an internal control because of an observed preferential use of this leg following BTX injection. Therefore, mice receiving the same volume of saline (0.9%) were used as study control (saline). After three weeks, the maximum effect of muscle disuse on bone loss was expected and therefore, half of the mice in both BTX and saline groups were euthanized (unloading phase, weeks 17 - 19, **Figure 1A**). To allow restoration of muscle function, the remaining mice were monitored until euthanasia (remobilization phase, weeks 20 - 35, **Figure 1A**). Femurs were dissected free of attached soft tissue and stored at -20°C in saline-moistened gauze while tibiae were stored at 4°C in 70% ethanol.

### Monitoring During Treatment

The general wellbeing of the animals was monitored daily, and food pellets were given in the bottom of the cage to ease feeding after BTX injection. The body weight was recorded at baseline (BL, 16 week), weekly after BTX administration for the first three weeks (17- 18- and 19 week) and at 27- and 35 weeks in the recovery group. All mice were pre-screened for a “zero” digit abduction score (DAS = 0) response and DAS assay was performed after BTX injection at days 1, 2, 4, 7, 10, 14 and 21 as described previously (27). Briefly, mice are suspended by their tails to elicit a characteristic startle response in which the animal extends its hind-limbs and abducts its hind digits. The degrees of digit abduction are scored on a five-point scale (0 = normal to 4 = maximum reduction in digit abduction) to measure the muscle weakening effectiveness.

### Mouse Skeletal Analysis and Bone Strength Measurements

To quantify bone mineral density (BMD, grams/cm^2^) of the right femur, dual energy x-ray absorptiometry (DXA) was performed after 3- and 19- weeks after BTX injection using a PIXImus DXA densitometer (Lunar Corporation, Madison, WI). Additional parameters assessed were bone mineral content (BMC, grams) and bone area (cm^2^), analyzed using PIXImus software. All scans were performed after rehydration of the femurs in a saline solution at room temperature overnight. After the scan, femur was placed on an Electropuls E1000 (Instron, High Wycombe, United Kingdom) for *ex vivo* biomechanical bone strength measurements. First, femur was placed axially using a customized fitting and an anvil was lowered onto the femoral head until fracture of the neck. Hereafter a three-point-bending test was performed on the femurs placed horizontally on two anvils (distance of 7 mm with mid-femur in the center and lowering a third anvil until deflection of the midshaft. For both measurements, a constant speed of 1 mm/minute and the maximum load was recorded.

### Micro-CT

For the microarchitectural analysis of the right tibiae, the X-ray source was operated at 70 kV and 114 µA with a 0.5- mm aluminum filter (vivaCT 40, Scanco Medical A, Brüttisellen, Switzerland). Images were reconstructed at a size of 10.5*10.5*10.5 μm^3^ voxels (2048*2048*2048 pixels) with 32-bit-gray-levels. Bone region of interest was defined as 1.26 mm-thick trabecular abundant region which was 0.5 mm lower than the growth plate. Cortical bone analysis was along the tibial midshaft and included BMD (grams of hydroxylapatite per cubic centimeter, mg HA/ccm), bone volume per total specimen volume (BV/TV, %), pore size (µm) and cortical thickness (µm). The trabecular bone-related quantitative parameters measured include BMD (mg HA/ccm), BV/TV (%), structure model index as an estimate of the rod or cone shape of trabeculae (SMI, -), trabecular number (1/mm), trabecular thickness (µm), trabecular separation determined as mean distance between trabeculae (µm), and trabecular degree of anisotropy (DA, -). All measurements are based on the guidelines from the American Society for Bone and Mineral Research (28).

### Bone Histomorphometry

Ten and two days before euthanasia, mice were injected IP with calcein (20 mg/kg) for labeling of newly formed bone. Right tibiae were dissected free of soft tissue at euthanasia and fixed in 70% ethanol at 4°C. After embedding in methyl methacrylate, bones were sectioned longitudinally through the frontal plane (7µm thick sections) using a Polycut E microtome (Leica Microsystems, Wetzlar, Germany). Sections were mounted on gelatin -coated slides and dried for 1 hour at 40°C before further treatment. Five sections from each bone remained unstained for quantification of mineralizing surfaces, calculated as the sum of doubly labeled plus half of singly labeled surfaces, as a percentage of bone surface (MS/BS, %). A minimum of five double labels were used to calculate mineral apposition rate (MAR, µm/day). Five sections from each tibia were stained with Goldner’s trichrome to determine eroded surface as a percentage of bone surface (ES/BS, %). All sections were analyzed using an Olympus BX51 microscope attached to an image analyzer (C.A.S.T. GRID system from Olympus Denmark 2000). Primary histomorphometric indices of bone formation are based on the American Society for Bone and Mineral Research histomorphometry nomenclature (29).

### Osteoblast and Osteoclast Isolation and Differentiation *In Vitro*

Tissue culture reagents were purchased from Thermo Fisher Scientific (Roskilde, Denmark); unless otherwise mentioned. Osteoblasts were differentiated *in vitro* from precursors obtained from long bones of 8-week-old female WT and KO littermates according to published protocols (30). Briefly, cells were grown to confluency in T25 flasks, before being harvested and densities adjusted for assays as required. Growth conditions were 37°C, 5% CO_2_ in culture media (Ob-CM) (MEM without Phenol Red supplemented with 10% Fetal Bovine Serum + 1% GlutaMax + 1% Pen/Strep) with media changed every 2 days. Precursors were seeded at a density of 10^5^ cells/mL in Ob-CM for 9 days. The ability of metabolically intact cells to cleave tetrazolium salt (WST-1) (Sigma-Aldrich, Soeborg, Denmark) into formazan dye representing cell viability and, staining for membrane alkaline phosphatase (ALP) as a marker for osteoblast maturation, were evaluated. In parallel cultures, ALP activity was measured by hydrolysis of p-nitrophenyl phosphate (pNPP) to p-nitrophenol (a chromogenic product). One unit of activity is defined as hydrolysis of 1 nmole of pNPP in one minute at pH 7.4 and standard reaction conditions. ALP activity was normalized to the amount of DNA per sample as determined by PicoGreen assay (Excitation at 480 nm/Emission at 520 nm) (Sigma-Aldrich, Soeborg, Denmark). For mineralization assay, precursors were cultured till confluency (approx. 14 days), and Ob-CM was supplemented with 50 μg/ml L-ascorbic acid phosphate (Wako Chemicals USA) and 10 nM Dexamethasone (Sigma-Aldrich) for a further 21 days. In the last 72 hours, 10 mM β-glycerophosphate (Sigma-Aldrich) was added and nodules quantified after Alizarin Red staining (AR-S, pH 4.2). Osteoclasts were derived from splenic precursors from 8-week-old female WT and KO littermates by gradient isolation on Histopaque-1077 according to the published protocols (31). Briefly, 10^6^ precursors were added in a 96 well and differentiated in the presence of 30 ng/ml rmM-CSF (R&D Systems, Boston, USA) and 50 ng/ml rmRANKL (R&D Systems). Growth conditions were 37°C, 7% CO_2_ in culture media (Oc-CM) (αMEM with 10% Fetal Bovine Serum + 1% GlutaMax + 1% Pen/Strep) with media changed every 2 days. Multinucleated osteoclasts were determined after 7 days by fixing cells in 10% formalin and staining for tartrate-resistant acid phosphatase (TRAP) (Sigma-Aldrich). Counterstaining with Gill’s hematoxylin was used to highlight nuclei and osteoclasts were defined as TRAP-positive cells with 3 or more nuclei. For osteoclast function, area of the resorption pits on the entire disc was quantified, when 10^6^ splenic precursors from both WT and KO mice were differentiated in Oc-CM supplemented with 30 ng/ml rmM-CSF and 50 ng/ml rmRANKL, USA) for 14 days, using ImagePro (Media Cybernetics, Inc. Rockville).

### *P2ry2* Gene Expression

All tissue samples were snap frozen after collection and were weighed equally before homogenization on dry ice. To determine *P2ry2* gene expression, DNA was extracted by tissue lysis (pH 12) genotyped using a triple primer PCR. Two different forward primers (5′-GTCACGCGCACCCTCTACTA-3′ and 5′-GGGGAACTTCCTGACTAGGG-3′) to identify the WT and the inserted neo-cassette in the disrupted allele respectively, were combined with the common reverse primer (5′-GTCGGGTGGCACTGCCTTTCT-3′). After 35 amplification cycles, the product was neutralized (pH 5) and run on 2% agarose to visualize WT (551 bp product), KO (700 bp product) and Het (700 bp and 551 bp products) mice. To determine *P2ry2* transcript expression, total RNA was isolated using RNeasy Minikit (QIAGEN Nordic – Copenhagen, Denmark) as per the manufacturer’s instructions. Total RNA (2 μg) was used for cDNA synthesis using the High Capacity cDNA Reverse Transcription kit (Applied Biosystems, Thermo Fisher Scientific) using Multiscribe Reverse Transcriptase and random primers and 1 μl of cDNA was used for end point PCR using HotStarTaq DNA Polymerase. Primer Blast (https://www.ncbi.nlm.nih.gov/tools/primer-blast/) was used to design two sets of forward and reverse primers on the *P2ry2* gene between 552 bp – 1149 bp corresponding to the region of the targeting vector used to create the gene knock out. *P2ry2* primer set 1, designed to amplify from 676 bp to 1065 bp: Forward 5′-TCAAACCGGCTTATGGGACC-3′, Reverse 3′-GTCGTCACTGCTGACTGACA-5′ generating a 389 bp product; *P2ry2* primer set 2, designed to amplify from 612 bp to 857 bp: Forward 5′-CTGCTTTTTGCTGTGCCCTT-3′, Reverse 3′-ATGTTGATGGCGTTGAGGGT-5′ generating a 245 bp product. Template loading was controlled using mGAPDH (Forward 5′-TTGAAGGTTGGAGCCAAACG-3′, Reverse 5′-TCATACCAGGAAATGAGC-3′, 587 bp product) and bone cell differentiation was confirmed using the sequences for OB- specific (mCol1A: Forward 5′-CTTCACCTACAGCACCCTTGTG-3′, Reverse 5′-GATGACTGTCTTGCCCCAAGTT-3′, 67 bp product and mRunX Forward 5′-GCGTATTTCAGATGATGACA-3′, Reverse 5′-TACCATTGGGAACTGATAGG-3′, 367 bp product) and OC- specific (mNF-κB/RANK: Forward 5′-TTTGTGGAATTGGGTCAATGAT-3′, Reverse 5′-ACCTCGCTGACCAGTGTGAA-3′, 249 bp product and mCathK: Forward 5′-ACGGAGGCATCGACTCTGAA-3′, Reverse 5′-GATGCCAAGCTTGCGTCGAT-3′, 200 bp product). All primers were ordered through Eurofins and used at 0.4 μM final concentration. Sanger sequencing (Eurofins Genomics Germany GmbH) was performed on the amplified PCR products using the forward primer 5’- TCAAACCGGCTTATGGGACCAC-3’ and sequences were aligned using DNASTAR Lasergene (trial version, DNASTAR, Wisconsin USA).

### Intracellular Calcium Measurements in Primary Cells

Intracellular release of calcium ([Ca^2+^]_i_) was determined as described previously (32). Cells were cultured for 2 days before loading with Fluo-4 AM (4 µM containing 50% v/v Pluronic F-127) dissolved in experiment medium (Hanks Buffer containing 20 mM HEPES, 2 mM CaCl_2_, 0.5 mM MgCl_2_, 2.5 mM Probenecid, pH 7.4) in the dark for 1 hour at 37°C/5% CO_2_. Cells were rinsed to remove excess Fluo-4 AM and equilibrated for 10 minutes before recording baseline fluorescence (Excitation/Emission wavelengths 485/520 nm, respectively). Agonist dissolved in experiment media was introduced after 30 s and area under the curve was calculated for the curve generated for the duration of 120 s. A calcium ionophore A23187 (50 μM), was used to saturate Fluo-4 with free Ca^2+^ at the end of each measurement. Cells with injections of only experiment medium were used as no agonist control.

### Statistics

Multifactorial analysis of variance (two- way ANOVA) was used to assess the influence of P2Y_2_ receptor deletion on the intervention with BTX where genotype (effect of KO when comparing WT vs KO) and treatment (effect of BTX when comparing saline vs BTX) are the two variables. *Post-hoc* Tukey’s multiple comparisons test was used to determine significant values as indicated. Percent differences are reported as the difference between BTX and saline control relative to the saline control such that the negative values reflect BTX related bone loss. Data was tested for normality using the Kolmogorov-Smirnov test and statistical significance between genotypes or treatment effects was determined using unpaired parametric t-test or non-parametric Mann-Whitney test. *In vitro* experiments were performed with littermate WT and KO pairs and shown as connecting lines, and results shown are obtained from at least three independent experiments. All data are expressed as mean ± SEM with significance tested using Prism 9.0 software (GraphPad, La Jolla, USA).

## Results

### Changes in KO Mice After Acute Muscle Paralysis-Induced Disuse of the Bone

Muscle paralysis, after BTX injection, was observed in both WT and KO mice from day 1 with the peak DAS value determined at day 4 in both BTX WT (3.31 ± 0.2) and BTX KO mice (3.05 ± 0.2) (**Figure 1B**). A slow and steady decay in DAS value was seen in both genotypes over a period of 21 days however, the DAS response diminished faster in KO BTX (1.74 ± 0.2) compared to the BTX WT (2.56 ± 0.3) at day 7 (*P* = 0.0333). Female BALB/cJ mice weighed 23.51 ± 0.18 g at baseline (BL, 16 weeks of age). Compared to the saline injected mice, both BTX WT and BTX KO mice showed significant body weight reduction after one week until the end of the 3- week observation period (*P* < 0.05) (**Figure 1C**). However, compared to the baseline a body-weight reduction after 17- (6.06% ± 2.1, *P* = 0.0441), 18- (7.69% ± 1.9, *P* = 0.0052) and 19 weeks (7.08% ± 1.6, *P* = 0.0123) was seen only in BTX KO mice; while, the reduction did not reach statistical significance in BTX WT mice. No changes in body weight were observed in the saline injected mice of either genotype. All measurement results are tabulated in **Table 1**.

**Table 1:** Quantitative data of DAS and Body weight in WT and KO mice injected with saline or BTX.

DAS values of mice during unloading phase, Mean ± SEM (n)				
Days (after BTX)	WT		KO	
	Saline	BTX	Saline	BTX
**1**	0 (15)	1.800 ± 0.28 (15)	0 (18)	1.789 ± 0.26 (19)
**2**	0 (16)	3.063 ± 0.17 (16)	0 (19)	3.053 ± 0.22 (19)
**4**	0 (16)	3.313 ± 0.19 (16)	0 (19)	3.053 ± 0.18 (19)
**7**	0 (16)	2.563 ± 0.30 (16)	0 (19)	**1.737 ± 0.23 (19)^a^**
**10**	0 (16)	1.375 ± 0.15 (16)	0 (19)	1.263 ± 0.10 (19)
**14**	0 (16)	1.250 ± 0.14 (16)	0 (19)	1.211 ± 0.16 (19)
**21**	0 (16)	0.875 ± 0.09 (16)	0 (19)	0.842 ± 0.09 (19)
**Body Weight (grams) of mice during the unloading phase, Mean ± SEM (n)**				
**Age (Weeks)**	**WT**		**KO**	
	**Saline**	**BTX**	**Saline**	**BTX**
**BL**	0 ± 1.46 (16)	0 ± 1.31 (16)	0 ± 1.46 (19)	0 ± 1.96 (18)
**17**	2.104 ± 1.74 (16)	**-6.249 ± 1.82 (16) ^a^**	2.967 ± 1.26 (19)	**-6.057 ± 2.10 (19) ^b,d^**
**18**	2.156 ± 1.39 (16)	**-6.274 ± 1.85 (16) ^a^**	3.125 ± 1.52 (19)	**-7.691 ± 1.87 (19) ^b,d^**
**19**	3.594 ± 1.52 (16)	**-5.520 ± 1.58 (16) ^a^**	3.665 ± 1.81 (19)	**-7.077 ± 1.63 (19) ^b,d^**
**Body Weight (grams) of mice in the remobilization phase, Mean ± SEM (n)**				
**Age (Weeks)**	**WT**		**KO**	
	**Saline**	**BTX**	**Saline**	**BTX**
**BL**	0 ± 1.34 (9)	0 ± 1.60 (10)	0 ± 2.11 (10)	0 ± 1.25 (9)
**17**	1.629 ± 1.82 (9)	**-5.950 ± 2.56 (10) ^a^**	2.228 ± 1.75 (10)	**-5.318 ± 1.72 (10) ^b^**
**18**	1.628 ± 1.07 (9)	**-6.577 ± 2.21 (10) ^a^**	2.564 ± 2.15 (10)	**-7.206 ± 1.19 (10) ^b^**
**19**	3.951 ± 1.56 (9)	**-6.742 ± 2.13 (10) ^a^**	4.580 ± 2.76 (10)	**-7.413 ± 1.03 (10) ^b^**
**27**	**9.994 ± 2.21 (9) ^c^**	3.870 ± 2.59 (10)	7.730 ± 2.03 (10)	4.748 ± 2.03 (10)
**35**	**9.251 ± 2.01 (9) ^c^**	6.367 ± 2.36 (10)	**10.418 ± 2.18 (10) ^d^**	**10.621 ± 2.69 (10) ^d^**

DAS response was measured at time points indicated. Value are means ± SEM (n = the number of mice used in the group). P-values < 0.05 are highlighted in bold where a = significance from BTX WT. Body weight is calculated as percentage change from baseline (BL, 16 week of age) = (Weight at age _mean_ – Weight at BL_mean_)/Weight at BL_mean_ × 100. P-values < 0.05 are highlighted in bold where significance from saline (a = WT, b = KO); and significance from BL (c = WT, d = KO).

A profound loss in the BMD, BMC and area of the femurs, excised 3 weeks after injection with BTX, was seen irrespective of the genotype (**Figure 1D**). Compared to the saline injected mice, the reduction in BMD was 10.27% ± 1.0 (*P* < 0.0001) in BTX WT versus 12.70% ± 0.88 in BTX KO (*P* < 0.0001); BMC was 15.57% ± 1.7 in BTX WT (*P* < 0.0001) versus 20.60% ± 2.1 in BTX KO (*P* < 0.0001); and, bone area was 5.99% ± 1.5 in BTX WT (*P* = 0.0084) versus 9.32% ± 1.7 in BTX KO (*P* < 0.0001) (**Table 1**). However, the BTX- induced bone deterioration was similar between the WT and KO mice. Furthermore, relative to the saline injected mice, the maximum load at the femur neck (metaphysis) was significantly reduced in both BTX WT (36.55% ± 3.4, *P* < 0.0001) and BTX KO mice (36.90% ± 2.3, *P* < 0.0001) (**Figure 1E**) with no difference in the reduction between the WT and KO mice. The measured maximum load at the femur diaphysis after BTX injection was significantly reduced in BTX KO (11.20% ± 2.3, *P* = 0.0005) but not in BTX WT mice (7.31% ± 2.8, P=0.0645) compared to their respective saline injected controls. However, the reduction in the femoral diaphyseal strength was not different between the WT and KO mice. All measurement results are tabulated in **Table 2**.

**Table 2:** Quantitative data of femoral DXA and bone strength parameters in WT and KO mice injected with saline or BTX.

Femur DXA after 3 weeks of BTX injection					
	WT		KO		
	Mean ± SEM (n)	%	Mean ± SEM (n)	%	P-value
**BMD (grams/cm^2^)**					
Saline	0.05246 ± 0.0005 (18)		0.05249 ± 0.0004 (23)		0.9644
BTX	0.04707 ± 0.0005 (15)	**-10.27 ^a^**	0.04582 ± 0.0004 (24)	**-12.70 ^a^**	0.0847
**BMC (grams)**					
Saline	0.01888 ± 0.0004 (18)		0.01909 ± 0.0003 (23)		0.6394
BTX	0.01594 ± 0.0003 (15)	**-15.57 ^a^**	0.01516 ± 0.0004 (24)	**-20.60 ^a^**	0.0970
**Bone Area (cm^2^)**					
Saline	0.3603 ± 0.005 (18)		0.3641 ± 0.004 (23)		0.5633
BTX	0.3387 ± 0.006 (15)	**-5.99 ^a^**	0.3302 ± 0.006 (24)	**-9.32 ^a^**	0.1967
**Femur bone strength after 3 weeks of BTX injection**					
	**WT**		**KO**		
	**Mean ± SEM (n)**	**%**	**Mean ± SEM (n)**	**%**	**P-value**
**Metaphysis (N)**					
Saline	14.60 ± 0.2 (15)		13.71 ± 0.3 (15)		**0.0451**
BTX	9.263 ± 0.5 (6)	**-36.55 ^a^**	8.653 ± 0.3 (15)	**-36.90 ^a^**	0.9360
**Diaphysis (N)**					
Saline	17.30 ± 0.4 (15)		17.85 ± 0.3 (18)		0.2966
BTX	16.03 ± 0.5 (12)	-7.31	15.85 ± 0.4 (19)	**-11.20 ^a^**	0.3002
**Femur DXA after 19 weeks of BTX injection**					
	**WT**		**KO**		
	**Mean ± SEM (n)**	**%**	**Mean ± SEM (n)**	**%**	**P-value**
**BMD (grams/cm^2^)**					
Saline	0.05536 ± 0.0008 (9)		0.05525 ± 0.0007 (10)		0.9179
BTX	0.05081 ± 0.0009 (10)	**-8.22 ^a^**	0.05132 ± 0.0004 (10)	**-7.11 ^a^**	0.5542
**BMC (grams)**					
Saline	0.02343 ± 0.0006 (9)		0.02256 ± 0.0006 (10)		0.3657
BTX	0.01970 ± 0.0006 (10)	**-15.91 ^a^**	0.01968 ± 0.0004 (10)	**-12.77 ^a^**	0.3660
**Bone Area (cm^2^)**					
Saline	0.4239 ± 0.007 (9)		0.4085 ± 0.008 (10)		0.1712
BTX	0.3860 ± 0.008 (10)	**-8.94 ^a^**	0.3860 ± 0.008 (10)	-5.51	0.2100
**Femur bone strength after 19 weeks of BTX injection**					
	**WT**		**KO**		
	**Mean ± SEM (n)**	**%**	**Mean ± SEM (n)**	**%**	**P-value**
**Metaphysis (N)**					
Saline	14.35 ± 0.5 (7)		14.74 ± 0.5 (9)		0.5783
BTX	12.28 ± 0.6 (10)	**-14.40 ^a^**	11.54 ± 0.3 (6)	**-21.72 ^a^**	0.4278
**Diaphysis (N)**					
Saline	21.26 ± 1.5 (9)		20.94 ± 0.9 (9)		0.8577
BTX	18.16 ± 0.9 (10)	-14.58	19.05 ± 1.1 (10)	-9.03	0.7394

Analysis was done after all groups were euthanized. Value are means or means ± SEM (n = the number of mice used in the group) or calculated as percentage change from saline (% = (BTX_mean_ – Saline_mean_)/Saline_mean_ × 100). P-values < 0.05 are highlighted in bold to show significance from WT calculated using either Student’s unpaired t-test/Mann-Whitney test (effect of KO) or, 2-way ANOVA (effect of BTX and KO) or, a = significance from saline (%) calculated using unpaired t-test.

**Figure 2:**
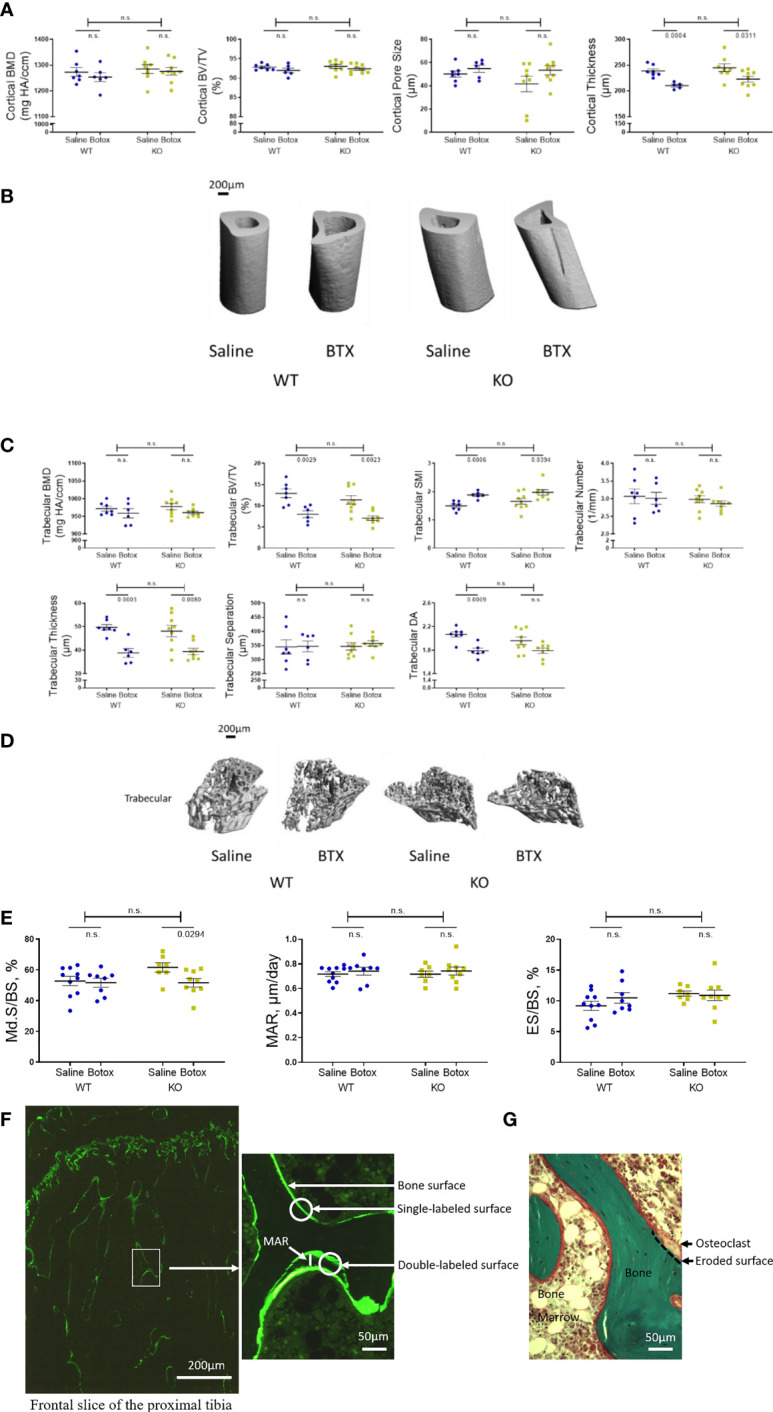
Tibial microstructural parameters determined after the unloading phase in both WT and KO at 3- weeks after BTX- induced muscle paralysis. **(A)** cortical bone mineral density (BMD, mg HA/ccm), bone volume (BV/TV, %), pore size (µm) and thickness (µm), and **(B)** representative images to show the changes in cortical indices. **(C)** Trabecular BMD (mg HA/ccm), BV/TV (%), structure model index (SMI, -), trabecular number (1/mm), trabecular thickness (µm), trabecular separation (µm), and trabecular degree of anisotropy (DA, -) and **(D)** representative images to show the changes in trabecular indices. **(E)** Histomorphometric indices to show to Md.S/BS (mineralized surfaces as percentage of bone surface), MAR (mineral apposition rate in µm/day) and ES/BS (eroded surface as percentage of bone surface) *in vivo*in both WT and KO mice at 3- weeks after BTX- induced muscle paralysis. **(F)** Illustrations of calcein labeled and **(G)** Goldner’s trichrome stained slices to quantify the histomorphometric indices. Statistical significance was tested with either Student’s unpaired t-test/Mann-Whitney test (effect of KO) or, 2-way ANOVA (effect of BTX and KO) with Tukey’s test for multiple comparisons. n.s., no significance. Data shows mean ± SEM of n = 6-7 mice (WT) and 8-9 mice (KO) for Micro-CT and, n= 8- 10 mice (WT) and 7-9 mice (KO) for histomorphometry.

Microarchitectural parameters assessed by µ-CT 3 weeks after BTX injection showed reduced cortical thickness in both WT (11.97% ± 2.6, *P* = 0.0004) and KO (8.98 ± 2.3, *P* = 0.0311) mice compared to the saline injected controls (**Figure 2A, B**). However, cortical BMD, BV/TV and pore size remained unchanged after BTX injections in both genotypes. Moreover, there was no difference in these cortical indices between the WT and KO mice after BTX. The microarchitecture of the trabecular compartment was substantially more affected at 3 weeks after BTX injection. Compared to the saline injected mice, the trabecular BV/TV was reduced by 38.01% ± 6.1 in BTX WT (*P* = 0.0029) versus 37.92% ± 4.7 in BTX KO (*P* = 0.0023); structure model index (SMI, a widely used measure for rods and plates in trabecular bone), was increased by 25.64% ± 3.3 in BTX WT (*P* = 0.0006) versus 19.32% ± 5.9 in BTX KO (*P* = 0.0394); thickness was reduced by 21.86% ± 3.7 in BTX WT (*P* = 0.0003) versus 27.12% ± 9.4 in BTX KO (*P* = 0.0080); and, degree of anisotropy (DA, a measure of trabecular orientation) were significantly reduced by 13.52% ± 2.2 in BTX WT (*P* = 0.0009) and approaching significance in BTX KO (8.64% ± 2.3, *P* = 0.0546). **Figure 2C, D**). However, the trabecular BMD, number and separation remained unchanged after BTX injections in both genotypes. Moreover, there was no difference in the changes of the trabecular compartment between the WT and KO mice after BTX. All measurement results are tabulated in **Table 3**.

**Table 3:** Quantitative data of tibial micro-CT and histomorphometric indices in WT and KO mice injected with saline or BTX.

Tibial Micro-CT after 3 weeks of BTX injection					
	WT		KO		
	Mean ± SEM (n)	%	Mean ± SEM (n)	%	P-value
**Cortical BMD (mg HA/ccm)**					
Saline	1273 ± 16.5 (7)		1285 ± 17.3 (8)		0.6275
BTX	1254 ± 17.3 (6)	-1.52	1275 ± 14.2 (9)	-0.73	0.6589
**Cortical BV/TV (%)**					
Saline	92.81 ± 0.3 (7)		93.02 ± 0. 5 (8)		0.7358
BTX	91.98 ± 0. 6 (6)	-0.89	92.38 ± 0. 4 (9)	-0.69	0.7711
**Cortical Pore Size (µm)**					
Saline	50.1 ± 0.3 (7)		41.6 ± 0.7 (8)		0.6340
BTX	54.8 ± 0.3 (6)	9.35	53.4 ± 0.4 (9)	28.40	0.1810
**Cortical Thickness (µm)**					
Saline	238.7± 4 (7)		244.6 ± 7 (8)		0.5146
BTX	210.1 ± 3 (6)	**-11.97 ^a^**	222.7 ± 6 (9)	**-8.98 ^a^**	0.3756
**Trabecular BMD (mg HA/ccm)**					
Saline	971.5 ± 6.74 (7)		977.5 ± 8.67 (9)		0.6116
BTX	958.8 ± 12.97 (6)	-1.31	960.5 ± 4.27 (8)	-1.74	0.7380
**Trabecular BV/TV (%)**					
Saline	12.93 ± 0.9 (7)		11.40 ± 1.0 (9)		0.3049
BTX	8.01 ± 0.8 (6)	**-38.01 ^a^**	7.08 ± 0.5 (8)	**-37.92 ^a^**	0.9905
**Trabecular SMI (-)**					
Saline	1.498 ± 0.06 (7)		1.656 ± 0.10 (9)		0.2344
BTX	1.882 ± 0.05 (6)	**25.64 ^a^**	1.976 ± 0.10 (8)	**19.32 ^a^**	0.4165
**Trabecular Number (1/mm)**					
Saline	3.065 ± 0.21 (7)		2.983 ± 0.10 (9)		0.7025
BTX	3.011 ± 0.17 (6)	-1.78	2.861 ± 0.08 (8)	-4.09	0.6885
**Trabecular Thickness (µm)**					
Saline	49.7 ± 0.1 (7)		48.1 ± 0.2 (9)		0.5900
BTX	38.8 ± 0.2 (6)	**-21.86 ^a^**	39.4 ± 0.1 (8)	**-27.12 ^a^**	0.6705
**Trabecular Separation (µm)**					
Saline	344.9 ± 25 (7)		346.8 ± 13 (9)		0.9454
BTX	346.9 ± 19 (6)	0.57	3574 ± 9 (8)	3.08	0.6680
**Trabecular DA (-)**					
Saline	2.069 ± 0.04 (7)		1.960 ± 0.07 (9)		0.2142
BTX	1.789 ± 0.04 (6)	**-13.52 ^a^**	1.791 ± 0.04 (8)	-8.64	0.1602
**Tibial Histomorphometric indices after 3 weeks of BTX injection**					
	**WT**		**KO**		
	**Mean ± SEM (n)**	**%**	**Mean ± SEM (n)**	**%**	**P-value**
**Md.S/BS (%)**					
Saline	52.81 ± 3.03 (10)		61.69 ± 3.06 (7)		0.0642
BTX	51.76 ± 2.85 (8)	-1.98	51.72 ± 2.73 (9)	**-16.15 ^a^**	0.0583
**MAR (μm/day)**					
Saline	0.717 ± 0.02 (10)		0.717 ± 0.02 (7)		0.5214
BTX	0.741 ± 0.32 (8)	3.33%	0.742 ± 0.32 (9)	3.54%	0.5686
**ES/BS (%)**					
Saline	9.19 ± 0.74 (10)		11.17 ± 0.43 (7)		0.0577
BTX	10.49 ± 0.85 (8)	14.12	10.90 ± 0.86 (9)	-2.43	0.1871

Analysis was done after 3 weeks of BTX injection when all groups were euthanized. Value are means or means ± SEM (n = the number of mice used in the group) as shown or calculated as percentage change from saline (% = (BTX_mean_ – Saline_mean_)/Saline_mean_ × 100). P-values < 0.05 are highlighted in bold to show significance from WT calculated using either Student’s unpaired t-test/Mann-Whitney test (effect of KO) or 2-way ANOVA (effect of BTX and KO) or, a = significance from saline (%) calculated using unpaired t-test.

Tibial histomorphometry analysis after 3 weeks of BTX injection showed reduced mineralized surfaces as percentage of bone surface (Md.S/BS) in BTX KO (16.15 ± 4.4, *P* = 0.0294) but not in BTX WT compared to the respective saline injected controls (**Figure 2E**). However, the eroded surface as percentage of bone surface (ES/BS) remained unchanged after BTX injection in both WT and KO mice (**Table 3**). Moreover, genotype did not influence the changes of neither of the bone formation parameters i.e. Md.S/BS or mineral apposition rate (MAR) nor the resorption parameter i.e. ES/BS, due to BTX.

### Changes in the Bone of KO Mice During the Restoration of Muscle Function

**Figure 3:**
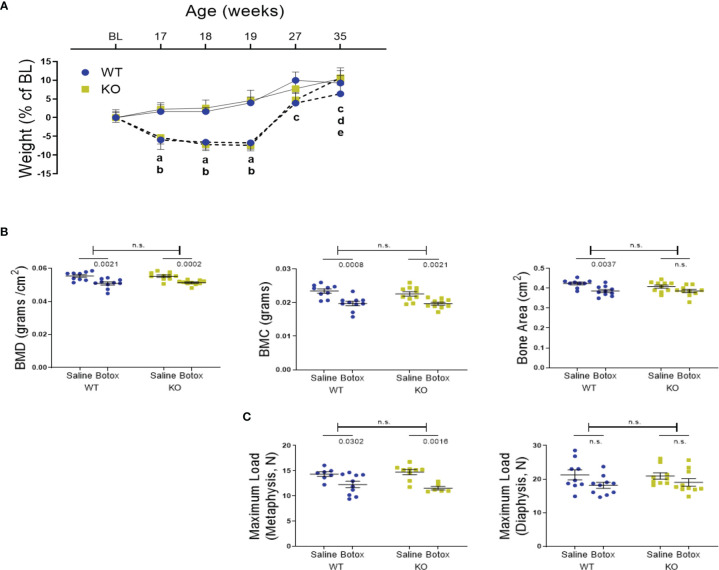
**(A)** Weight changes during the unloading and remobilization phase, expressed as a percentage change from baseline body weight (BL, 16-week old) in WT and KO mice injected with either saline (solid line) or BTX (dotted line). Significant body weight reduction after BTX (a, P < 0.05 in WT and b, P < 0.05 in KO) compared to the saline controls and, in compared to the BL (c, P < 0.05 in WT; d, P < 0.05 in KO and e, P < 0.05 in BTX KO). Statistical significance was tested using 2- way ANOVA with Tukey’s test for multiple comparisons. Data shows mean ± SEM of n = 9 - 10 mice (WT) and 10 mice (KO) in each treatment group. **(B)** DXA assessments of femoral bone mineral density (BMD, grams/cm^2^), bone mineral content (BMC, grams) and bone area (cm^2^) and, **(C)** femoral maximum load (N), at metaphysis and diaphysis in both WT and KO at 19- weeks after BTX- induced muscle paralysis. Statistical significance was tested with either Student’s unpaired t-test/Mann-Whitney test (effect of KO) or, 2-way ANOVA (effect of BTX and KO) with Tukey’s test for multiple comparisons. n.s., no significance. Data shows mean ± SEM of n = 9-10 mice (WT) and 10 mice (KO) for DXA and n= 7-10 mice (WT) and 6-10 mice (KO) for bone strength.

Next, we determined whether P2Y_2_ receptor played a role in bone recovery after restoration of muscle function. After a significant reduction in body weight of both WT BTX and BTX KO mice for the first 3- weeks of BTX injection, body weight was fully restored in both and not statistically significant from their saline counterparts (**Figure 3A**). A significant weight gain was seen at the end of the observation period (35- weeks) in BTX KO (10.62% ± 2.7, *P* = 0.0040) but not in BTX WT (6.37% ± 2.4) compared to the baseline (16- weeks). Additionally, saline injected WT mice show significant weight gain at 27 weeks (9.99% ± 2.2 g, *P* = 0.0112), 35 weeks (9.25% ± 2.0 g, *P* = 0.0246) and KO mice at 35 weeks (10.42% ± 2.2 g, *P* = 0.0036) compared to the baseline.

DXA analyses showed that the bone deterioration was not fully reversed after the recovery from muscle paralysis. Loss of BMD and BMC was still significant compared to the saline injected mice, in both genotypes after 19 weeks of BTX injection (**Figure 3B**). Femoral reduction in BMD was 8.22% ± 1.7 in BTX WT (*P* = 0.0021) versus 7.11% ± 0.84 in BTX KO (*P* = 0.0002); BMC was 15.91% ± 2.8 in BTX WT (*P* = 0.0008) versus 12.77% ± 1.9 in BTX KO (*P* = 0.0021). Compared to the saline injected controls, the femoral area was significantly reduced by 8.94% ± 1.8 in BTX WT (*P* = 0.0037) versus a reduction of 5.51% ± 2.0 in BTX KO (*P* = 0.0696) (**Table 2**). Furthermore, maximum load at the femur neck (metaphysis) was significantly reduced in both BTX WT (14.40% ± 4.5, *P* = 0.0302) and BTX KO mice (21.72% ± 2.3, *P* = 0.0016) (**Figure 3C**), relative to the saline injected mice. The measured maximum load at the femur diaphysis was reduced by 14.58% ± 4.2 in BTX WT and 9.03% ± 5.3 in BTX KO but was not statistically significant compared to their respective saline injected controls (**Table 2**). Genotype did not influence the bone recovery over the 16- week period, from muscle paralysis induced bone loss. All measurement results are tabulated in **Table 2**.

### Bone Cells in KO Mice

**Figure 4:**
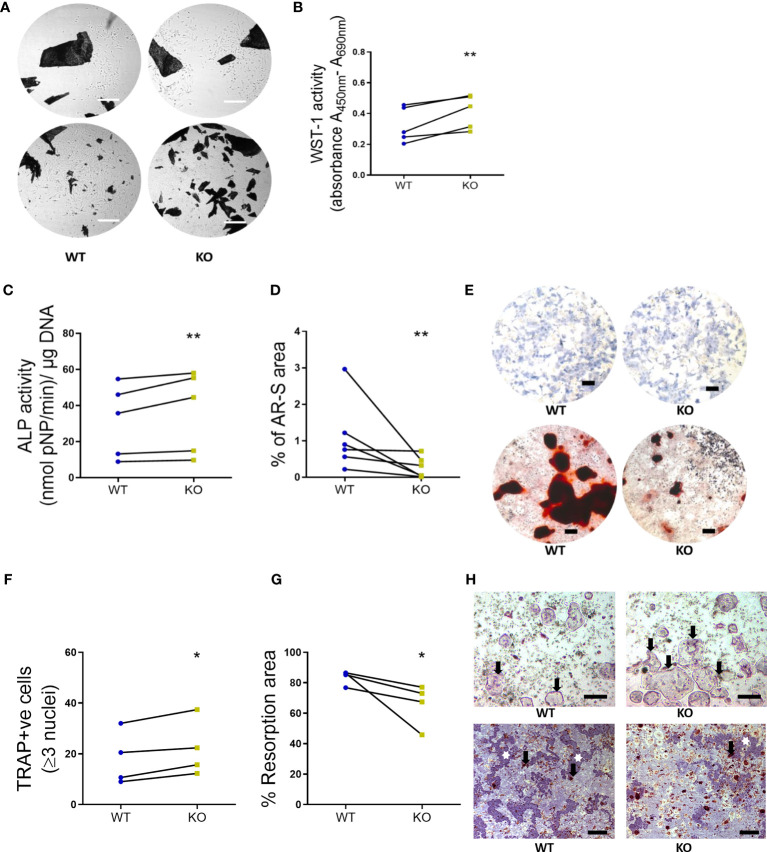
Primary bone cells were derived from 8-week-old WT and KO mice to determine the formation and functional characteristics. **(A)** Representative images to show no morphological differences between the unstained osteoblasts derived from long bone explants (dark spots represent the chopped bone pieces) at initial out-growth (top panel) and as confluent monolayers before subculture (bottom panel). Scale bar, 200 μm. **(B)** Osteoblast viability (WST-1 activity, **(C)** alkaline phosphatase activity (ALP activity, normalized to the amount of DNA per sample) and **(D)** bone nodule formation (% of AR-S stained area). **(E)** Representative images to illustrate the ALP staining (blue stain, top panel) and AR-S staining (brown stain showing mineralized bone structures, bottom panel) between WT and KO osteoblasts. Scale bar, 200 μm. **(F)** Osteoclast number (TRAP positive cells), **(G)** the resorption area excavated from the dentine substrate (expressed as a percentage of total dentine surface). **(H)** Representative images to illustrate the multinucleated TRAP positive osteoclasts (black arrows, top panel, scale bar, 50 μm) and resorption pits (white stars, bottom panel, scale bar, 200 μm) excavated by osteoclasts (black arrows, bottom panel) differentiated from the spleen of WT and KO littermate pairs. Statistical significance was tested with either Student’s unpaired t-test/Mann-Whitney test where *P-values < 0.05, **P-values < 0.01 show significance from WT. Data shows mean ± SEM of n = 5 littermate mice (osteoblasts) and n= littermate 4 mice (osteoclasts) repeat experiments with 6-10 replicate wells per experiment.

Primary bone cells were derived from 8-week-old skeletally mature KO female mice and the morphology, formation and functional characteristics were compared to littermate WT mice. Osteoblasts, derived from the bone marrow of KO mice, were not visually different from their WT counterparts (**Figure 4A**). However, KO osteoblasts viability was significantly higher at 9 days (1.31- fold, *P* = 0.0079) (**Figure 4B**) and displayed an increased alkaline phosphatase activity (1.15- fold, *P* = 0.0114) compared to the osteoblasts from WT mice (**Figures 4C, E**). On the contrary, the bone nodule formation was significantly lower in the KO compared to the WT osteoblasts (0.30- fold, *P* = 0.0022) (**Figures 4D, E**). Precursors from the spleens of KO and WT mice were used to determine osteoclast formation and function, by assessment of TRAP-positive cells and resorptive ability on a bone substrate, respectively. A higher number of TRAP-positive cells were generated from the KO precursors (1.27- fold, *P* = 0.0286) (**Figures 4F, H**) but, excavated a significantly reduced amount of total resorptive area, as measured from the dentine discs compared to the WT osteoclasts (0.79- fold, *P* = 0.0286) (**Figures 4G, H**).

### P2Y_2_ Receptor Expression in Bone Cells From KO Mice

**Figure 5:**
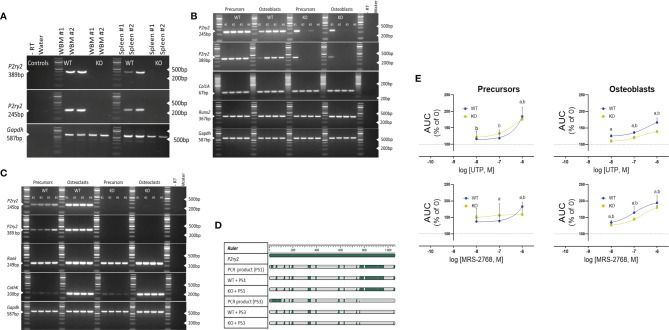
Expression of the P2Y_2_ receptor in **(A)** whole bone marrow (WBM) and spleen, **(B)** osteoblast precursors and osteoblasts and, **(C)** osteoclast precursors and osteoclasts. cDNA was reverse transcribed after tissue lysis and probed using the 2 different primer sets designed between 552 bp – 1149 bp of the *P2ry2* gene transcript (corresponding to the region of the targeting vector used to create the gene knock out). Products of 389 bp (corresponding to 676 bp -1065 bp) and 245 bp (corresponding to 612 bp to 857 bp) were amplified in osteoblast precursors and osteoblasts of KO mice. *Col1A* and *Runx2* were used as positive control for osteoblast- lineage and -differentiation, *RANK* and *CathK* were used as positive control for osteoclast-differentiation and *GAPDH* was used as sample loading control. **(D)** Alignment of the amplified PCR products (389 bp product = PS1 and 245 bp product = PS3) indicates matched regions (green) between the transcript from KO cells, WT cells and the *P2ry2* receptor gene sequence. **(E)** Intracellular release of [Ca^2+^]_i_ expressed as area under the curve (AUC) with increasing doses (10^-8^ - 10^-6^ M) of UTP (a potent agonist at the P2Y_2_ receptor) and MRS-2768 (a selective P2Y_2_ receptor agonist with no affinity for human P2Y_4_ or P2Y_6_ receptors) shows a functional response in the osteoblast precursors and osteoblasts from WT and KO mice. Statistical significance was tested with one-way ANOVA from no agonist control using (a, P < 0.05 in WT and b, P < 0.05 in KO. Data shows mean ± SEM of n = 3 repeat experiments with 3 replicates per dose of each agonist.

Rodent models generated with modified gene expression of purinergic receptors have greatly advanced bone research. However, the global deletion has been challenging as numerous reports demonstrate either the escape of gene deletion in the bone cells or presence of a transcript variant that may also be regulated by cell differentiation. We characterized the P2Y_2_ receptor expression in organs used to derive the bone cells, i.e. whole bone marrow and spleen for osteoblasts and osteoclasts respectively. These organs from the WT and KO mice were lysed to reverse - transcribe the cDNA and probed using the 2 different primer sets for the *P2ry2* gene transcript. The primers were designed between 552 bp – 1149 bp corresponding to the region of the targeting vector used to create the gene knock out. Bands corresponding to the *P2ry2* mRNA were detected in the whole bone marrow and spleen of WT mice but not in the whole bone marrow or spleen of the KO mice (**Figure 5A**). Furthermore, the *P2ry2* mRNA was detected in both, osteoblast precursors and mature osteoblasts as well as, the osteoclast precursors and mature osteoclasts from WT mice (**Figures 5B, C**). Surprisingly *P2ry2* mRNA was detected in osteoblastic precursors and mature osteoblasts in two out of four KO mice. The amplified PCR products were sanger sequenced and the sequences detected from KO cells align with the original *P2ry2* gene and with the sequences obtained from the WT cells (**Figure 5D**). To address whether this detected transcript has any functional implications, we performed [Ca^2+^]_i_ assays with increasing doses (10^-8^ - 10^-6^ M) of UTP (a potent agonist at the P2Y_2_ receptor) and MRS-2768 (a selective P2Y_2_ receptor with no affinity for human P2Y_4_ or P2Y_6_ receptors) (33). Precursors and mature osteoblasts from KO mice were loaded with high- affinity Ca^2+^ indicator and [Ca^2+^]i response were measured as a percent increase in Ca^2+^ signal (area under the curve) of non- stimulated cells (no agonist) (**Figure 5E**). Osteoblast precursors from WT mice showed agonist-evoked [Ca^2+^]i responses at the highest agonist concentrations (10^-6^ M UTP, 185%; *P* = 0.0027 and 10^-6^ M MRS-2768, 183%; *P* = 0.0031). [Ca^2+^]_i_ responses were also seen in osteoblast precursors from KO mice however, of a lower magnitude compared to the WT cells at the same concentration of the agonists (10^-6^ M UTP, 176%, *P* = 0.0005 and 10^-6^ M MRS-2768, 159%, *P* = 0.0460) (**Figure 5D**). Similarly, both agonists evoked [Ca^2+^]_I_ responses in osteoblasts of WT and KO mice however, the magnitude of responses were lower in KO osteoblasts for both agonists (10^-6^ M UTP, 167% in WT *versus* 139% in KO; 10^-6^ M MRS-2768, 184% in WT *versus* 180% in KO). Since MRS-2768 is also a triphosphate and therefore accessible to ectonucleotides (similar to UTP), these agonists likely evoked [Ca^2+^]i responses by generating nucleotides without the involvement of P2Y_2_ receptor. We performed a functional assay in the coronary arteries of WT and KO mice using MRS-2768 and UTPγS trisodium salt (selective P2Y_2/4_ agonist) (**Supplementary Figure S1**). MRS-2768 did not elicit a dilation response in the WT or KO coronary artery up to the tested concentration of 30 µM, unlike UTPγS where 50% vasodilation was seen in WT but not in KO, confirming a functional difference in the endothelium of the KO and WT.

## Discussion

In this study, we investigate the involvement of the P2Y_2_ receptor in muscle paralysis-induced bone loss. The main findings are: 1) a single injection with BTX causes rapid muscle dysfunction leading to profound bone deterioration, 2) P2Y_2_ receptor KO mice are not protected against the muscle paralysis- induced bone degradation, 3) Bone quality is not fully reversed after 19 weeks of BTX exposure and P2Y_2_ receptor has an inconsequential role in the bone recovery in our mouse model and, 4) primary osteoblasts and osteoclasts from P2Y_2_ KO mice show an increased formation of the bone cells however, the bone cells display a compromised function compared to the bone cells from the WT littermates.

BTX has a rapid muscle-paralyzing activity as an increase in the mean DAS value is seen at day 1 after the injection (**Figure 1B**). While there is no difference in kinetics of onset of DAS values between WT and KO mice, paresis is restored quicker in KO mice (day 7), supporting the role of P2Y_2_ receptor in delaying the rehabilitation after muscle weakening and paralysis. An anti- regenerative potential of the P2Y_2_ receptor during tissue injury and remodeling is previously described with initiation of a pro-fibrotic response after UTP-induced activation of skeletal muscle and cardiac fibroblasts (34, 35). The general health of the mice is significantly affected as seen by the body weight reduction of both genotypes for the first 3 weeks after BTX injection (**Figures 1C**, **3B**); but BTX KO experience a more drastic weight loss compared to BTX WT. The dramatic weight loss in female mice is not uncommon after BTX (36), and is followed by a gradual reversal and a complete restoration after 12 weeks. Similar body weight restoration is also seen during the recovery period in our BALB/cJ mice, regardless of the genotype, but BTX KO mice gain significantly more weight by the end of the observation period compared to their baseline body weight (**Figure 3B**). Moreover, a comparison with the saline injected control mice shows significant weight gain in P2Y_2_ receptor KO mice at 35 weeks but at a slower rate than the WT mice (already at 27 weeks). Since basal activation of P2Y_2_ receptor is essential for adipose tissue metabolism (37) and in promoting diet- induced obesity (38), an aberrant lipid metabolism is indicated in these KO mice. Moreover, body weight changes highlight a potential role of P2Y_2_ receptor in age- related changes, since mice after 26 weeks are skeletally developed (39) and are considered as reference group in aging studies (40). Our current data shows that knocking out the P2Y_2_ receptor quickens the restoration of muscle activity and the body mass regain from BTX-induced muscle paralysis.

Rodent models previously used to investigate the role of the P2Y_2_ receptor in bone physiology, indicate functional defects in both the osteoblasts and osteoclasts. For instance, an increase in BMC in hind limbs of 8 week - old P2Y_2_ receptor KO male mice is attributed to an anti- osteogenic potential of P2Y_2_ receptor activation (23). An age-related (4- through 24- weeks) increase in trabecular bone mass, due to a defective osteoclastic resorption, is also reported in these mice (14). Studies from another group describe compromised differentiation, function and diminished ERK1/2 phosphorylation in osteoblasts from P2Y_2_ receptor KO mice (25). Since ERK activation phosphorylates Runx2, a major transcriptional regulator of osteoblast-specific gene expression, a reduction in trabecular bone volume at 8 weeks and cortical strength at 17- weeks is reported in the P2Y_2_ receptor KO mice. Our data indicates skeletal fragility in KO mice as seen with the reduced strength of their femoral metaphysis at 16-weeks, compared to the WT mice (P= 0.0451, **Table 2**). Given the interplay between response to mechanical behavior and bone structure to deliver bone strength, a fragile bone structure is compatible with the observation of an impaired osteoblast function in primary cells from our P2Y_2_ receptor KO mice (**Figure 4D**). A reduced osteoblastic mineralization, despite a higher number of osteoblasts generated *in vitro*, strengthens the notion that P2Y_2_ receptor signaling is essential for normal osteoblast function. Since the P2Y_2_ receptor transmits the mechanically- induced signals between neighboring osteoblast-like cells (41), evokes mechanical and UTP- stimulated ATP release from osteocytes (12, 15) and osteoblasts (14); a lacking mechanical signaling system may explain the impaired osteoblast function. Additionally, a synergistic stimulus such as that from parathyroid hormone - mediated ATP release (42, 43) may be compromised in the P2Y_2_ receptor deficient osteoblasts. We also see a downregulation of bone resorptive function in KO osteoclasts (**Figure 4G**), demonstrated by their compromised function reflected in the reduced excavation of dentine substrate in spite of a higher number of osteoclasts generated from KO (**Figure 4F**). Further studies could identify if the bone resorptive function can potentially be rescued by the addition of exogenous ATP as previously reported from P2Y_2_ receptor deficient osteoclasts (14).

There were no large differences in the bone phenotype of our P2Y_2_ receptor KO mice, and we challenged the bone metabolism to determine whether the P2Y_2_ receptor regulates bone remodeling during unloading of the bone. Profound bone degradation was observed in the femoral BMD, BMC, and area in KO mice after 3- weeks of BTX injection, as well as severe alterations in the tibial microstructural parameters were revealed (**Tables 2**, **3**). Cortical thickness, trabecular bone volume and trabecular thickness were significantly reduced in the KO mice after BTX- induced muscle paralysis. However, the BTX induced decline of the bone parameters was also seen in the WT littermates and therefore, independent of the P2Y_2_ receptor gene expression. The findings more reflect the mechanics of BTX on bone, as previous studies using BTX to paralyze the lower limb musculature in mice show a reduction in BMC, cortical and trabecular thickness and trabecular bone volume (36, 44). In our study the KO mice show a significant reduction in the femoral diaphyseal strength, which was less profound in the BTX WT mice, compared to the saline injected controls (**Table 2**). Even though the mechanism of bone loss with the use of BTX is primarily due to an increase in bone resorption (45), a hindered osteoblast response due to the uncoupling of bone remodeling could be expected. Indeed, the decreased formation in KO tibia after BTX (**Table 3**) implies an aberrant bone turnover however, the histomorphometric indices showed unchanged resorption. Additionally, a deterioration of trabecular anisotropy (DA) in WT due to BTX, but not as severe in the KO tibia, was measured in comparison to the respective saline injected controls (**Table 3**). DA indicates a change in the trabecular mechanical integrity during unloading (46), thus, the lacking mechano-response leading to weaking of the trabecular bone may be preserved in the absence of P2Y_2_ receptor.

Remobilization studies using BTX to induce bone degradation have shown limited recovery of the bone lost (36, 44). In our study, we provided a 16 week recovery period, 3 weeks after BTX-induced muscle paralysis. We expected to see a quicker recovery of bone in the KO mice compared to the WT mice based on a faster restoration of muscle activity and weight regain after BTX injection. Determination of bone quality by DXA shows femoral BMD, BMC and area were still significantly reduced in WT compared to the saline injected mice, after 19 weeks of BTX injection (**Table 2**). The lost bone quality is also maintained in KO mice with BMD and BMC still lower in the BTX injected femur. Comparison of microstructure after recovery period was prevented as the BTX WT tibia showed no remaining trabeculae and reflects both a higher sensitivity of unloading at another skeletal site and adaptation of the bone to a new strain environment. Overall, even though the bone deterioration is not fully recovered, the differences measured in KO mice show minimal recovery from bone loss at the end of the recovery period, compared to the recovery in WT mice.

The major limitation of our study was the lacking serial measurements of the bone quality after the restoration of the muscle function. These measurements would provide an insight into the rate of bone recovery in each genotype and identify whether full reversal of bone quality could be achieved with a longer recovery period. Moreover, despite an improvement of the muscle function it is possible that the muscle strength remained impaired in our mice. A decreased muscle strength would reduce the muscle contractile effects on the bone, thereby reducing the anabolic stimulus to the bone. In addition, P2Y_2_ receptor expression was detected at the mRNA level and, appears to mediate agonist- induced intracellular calcium response in a proportion of osteoblast precursors and mature osteoblasts (**Figure 5**). If the P2Y_2_ receptor expression *in vitro* is a representation of the *in situ P2ry2* gene expression, the role of P2Y_2_ receptor in mediating BTX induced unloading and subsequent reloading of the bone, could be under interpreted in our study.

In summary, we show a profound bone loss of bone in a model for BTX- induced muscle paralysis. However, our findings do not indicate a regulation of the P2Y_2_ receptor in the bone loss during the skeletal unloading or, in the bone recovery after restoration of muscle function in mice.

## Data Availability Statement

The original contributions presented in the study are included in the article/**Supplementary Material**. Further inquiries can be directed to the corresponding authors.

## Ethics Statement

The animal study was reviewed and approved by Dyreforsoegstilsynet, Copenhagen, Denmark. License number: BTX 2012-15-2934-00148.

## Author Contributions

AA conducted the research, analyzed and interpreted data, and wrote the manuscript; ME conducted the research, interpreted data, and edited the manuscript; KH conducted the research and edited the manuscript; NW, AG, MD, and HP provided vital materials, interpreted data, and edited the manuscript; NJ designed the research, interpreted data, and edited the manuscript. All authors contributed to the article and approved the submitted version.

## Funding

The project was funded by the Novo Nordisk Foundation grant #6811.

## Conflict of Interest

The authors declare that the research was conducted in the absence of any commercial or financial relationships that could be construed as a potential conflict of interest.

## Publisher’s Note

All claims expressed in this article are solely those of the authors and do not necessarily represent those of their affiliated organizations, or those of the publisher, the editors and the reviewers. Any product that may be evaluated in this article, or claim that may be made by its manufacturer, is not guaranteed or endorsed by the publisher.

## Supplementary Material

Supplementary Figure 1 Functional effect of P2Y_2_ receptor agonists MRS-2768 and UTPγS, on coronary arteries of WT and KO mice. As the osteoblasts derived from the whole bone marrow showed a positive response in the KO, we wanted to rule out the possibility of a ‘tissue’ specific variant or a differentiation dependent transcript. For this, pharmacological activity of agonists MRS-2768 and UTPγS was determined on coronary arteries of WT and KO mice as previously described (47). Briefly, mice were killed by decapitation and the heart was immediately excised and placed in ice cold oxygenated calcium free physiological saline solution (Ca^2+^- free PSS) composed of (in mmol/L): 119 NaCl, 4.7 KCl, 2.5 CaCl_2_, 25 NaHCO_3_, 1.17 MgSO_4_, 1.18 KH_2_PO_4_, 5.5 glucose, and 0.03 EDTA, pH 7.4. The left anterior descending artery was dissected in ice cold, oxygenated Ca^2+-^free PSS and mounted on a Mulvany–Halpern wire myograph (Danish Myo Technology, Denmark). The myographs were connected to a PowerLab Unit and responses were sampled in LabChart™ (ADInstruments, UK). The mounted artery segments were heated to 37°C, and buffer changed for PSS with calcium. After 15 min equilibration, the vessels were stretched to their optimal lumen diameter L1 = 0.9 × L100, where L100 is an estimate of the diameter of the vessel under a passive transmural pressure of 13.3 kPa on a 25 µm wire. Subsequently, the vessels were allowed to stabilize for 20 –30 min. During incubation procedure in the myograph, the vessels were kept at this standard tension. Endothelium function was determined by assessing the relaxation a concentration dependence curve for UTPγS or MRS2768 after a steady-state pre-contraction with U46619 (100 nmol/L). Maximal functional endothelium was tested by applying 10 -5 M carbachol. Endothelial function was defined as percent of the precontraction to baseline. MRS-2768 had no effect on the dilation of the coronary artery in WT mice up to the tested concentration of 30µM and UTPyS caused 50% vasodilation at 1µM in WT (* P-value < 0.05) but not in KO coronary artery.
