# Supplementary figures and images for: Absence of P2Y2 Receptor Does Not Prevent Bone Destruction in a Murine Model of Muscle Paralysis-Induced Bone Loss
Source: Front Endocrinol (Lausanne). 2022 May 26;13:850525. doi: 10.3389/fendo.2022.850525 (PMC9204296; doi:10.3389/fendo.2022.850525)

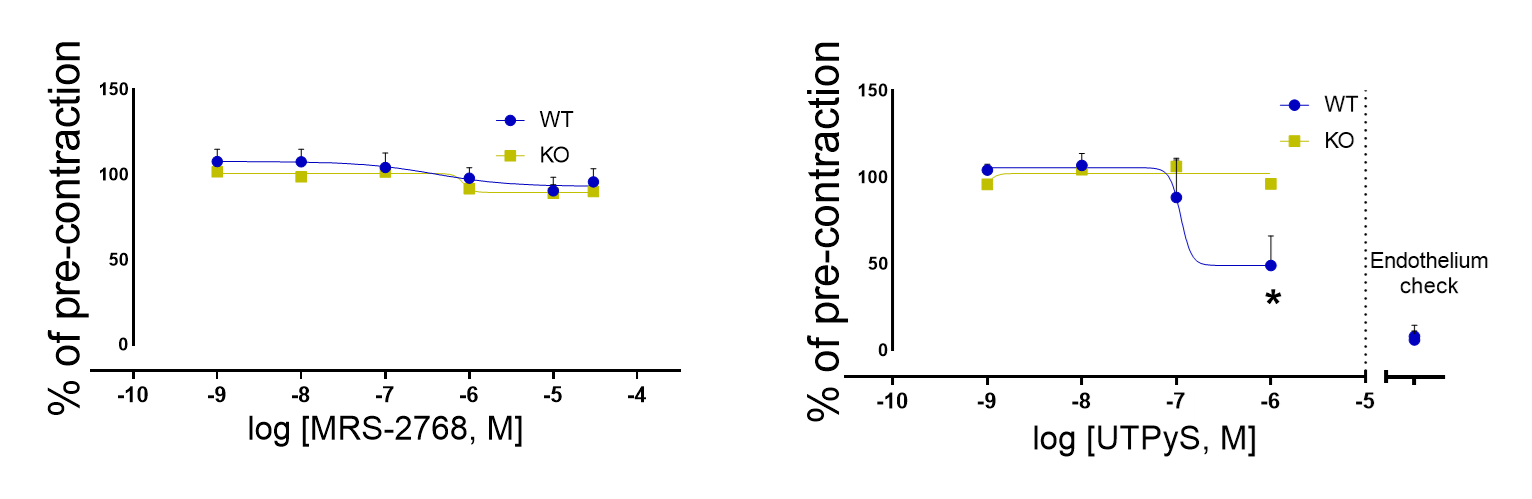

Supplement: Supplementary Figure 1 — Functional effect of P2Y2 receptor agonists MRS-2768 and UTPγS, on coronary arteries of WT and KO mice. As the osteoblasts derived from the whole bone marrow showed a positive response in the KO, we wanted to rule out the possibility of a ‘tissue’ specific variant or a differentiation dependent transcript. For this, pharmacological activity of agonists MRS-2768 and UTPγS was determined on coronary arteries of WT and KO mice as previously described (47). Briefly, mice were killed by decapitation and the heart was immediately excised and placed in ice cold oxygenated calcium free physiological saline solution (Ca2+- free PSS) composed of (in mmol/L): 119 NaCl, 4.7 KCl, 2.5 CaCl2, 25 NaHCO3, 1.17 MgSO4, 1.18 KH2PO4, 5.5 glucose, and 0.03 EDTA, pH 7.4. The left anterior descending artery was dissected in ice cold, oxygenated Ca2+-free PSS and mounted on a Mulvany–Halpern wire myograph (Danish Myo Technology, Denmark). The myographs were connected to a PowerLab Unit and responses were sampled in LabChart™ (ADInstruments, UK). The mounted artery segments were heated to 37°C, and buffer changed for PSS with calcium. After 15 min equilibration, the vessels were stretched to their optimal lumen diameter L1 = 0.9 × L100, where L100 is an estimate of the diameter of the vessel under a passive transmural pressure of 13.3 kPa on a 25 µm wire. Subsequently, the vessels were allowed to stabilize for 20 –30 min. During incubation procedure in the myograph, the vessels were kept at this standard tension. Endothelium function was determined by assessing the relaxation a concentration dependence curve for UTPγS or MRS2768 after a steady-state pre-contraction with U46619 (100 nmol/L). Maximal functional endothelium was tested by applying 10 -5 M carbachol. Endothelial function was defined as percent of the precontraction to baseline. MRS-2768 had no effect on the dilation of the coronary artery in WT mice up to the tested concentration of 30µM and UTPyS caused 50% vasodilation at 1µM i [file Image_1.tif]
